# Heat-Treated *Bifidobacterium longum* CECT-7347: A Whole-Cell Postbiotic with Antioxidant, Anti-Inflammatory, and Gut-Barrier Protection Properties

**DOI:** 10.3390/antiox10040536

**Published:** 2021-03-30

**Authors:** Patricia Martorell, Beatriz Alvarez, Silvia Llopis, Veronica Navarro, Pepa Ortiz, Nuria Gonzalez, Ferrán Balaguer, Antonia Rojas, Empar Chenoll, Daniel Ramón, Marta Tortajada

**Affiliations:** Archer Daniels Midland, Nutrition, Health&Wellness, Biopolis S.L. Parc Scientific Universitat de València, C/Catedrático Agustín Escardino Benlloch, 9, Paterna, 46980 Valencia, Spain; patricia.martorell@adm.com (P.M.); beatriz.alvarez@adm.com (B.A.); silvia.llopis@adm.com (S.L.); Veronica.NavarroBarrera@adm.com (V.N.); josefa.ortiz@adm.com (P.O.); nuria.gonzalez@adm.com (N.G.); ferran.balaguer@adm.com (F.B.); antonia.rojas@adm.com (A.R.); maria.chenoll@adm.com (E.C.); daniel.ramonvidal@adm.com (D.R.)

**Keywords:** *Bifidobacterium*, postbiotic, probiotic, *Caenorhabditis elegans*, anti-inflammatory, gut-barrier

## Abstract

Non-viable preparations of probiotics, as whole-cell postbiotics, attract increasing interest because of their intrinsic technological stability, and their functional properties, such as immune system modulation, gut barrier maintenance, and protection against pathogens. However, reports on *Bifidobacteria*-derived postbiotics remain scarce. This study aims to demonstrate the functional properties of a heat-treated (HT), non-viable, *Bifidobacterium longum* strain, CECT-7347, a strain previously selected for its anti-inflammatory phenotype and ability to improve biomarkers of intestinal integrity in clinical trials. The study used the nematode *Caenorhabditis elegans* and HT-29 cell cultures as eukaryotic model systems. Our results show that HT-CECT-7347 preserves the capacity to protect against oxidative stress damage, while it also reduces acute inflammatory response and gut-barrier disruption, and inhibits bacterial colonization, by activating pathways related to innate immune function. These findings highlight the interest of the ingredient as a novel postbiotic and pave the way to broaden the range of HT-CECT-7347 applications in gut health.

## 1. Introduction

Probiotics, defined by the World Health Organization decades ago as live microorganisms that, when administered in adequate amounts, confer a health benefit on the host, and generally understood as beneficial bacteria for host health, are currently widespread in food and beverages, including infant formulas, feed, and nutritional supplements [1,2,3]. Probiotics have been shown to balance and restore the gut microbiome, confer protection against pathogen infections, modulating immune function, and maintain intestinal barrier integrity, while showing therapeutic and prophylactic potential for a variety of conditions related to gastrointestinal health, but also extra-intestinal disorders, some of which are correlated with gut dysbiosis [4,5].

As greater knowledge is accrued on the mechanisms through which probiotic microorganisms exert their action, a variety of extracellular compounds have been identified as key mediators in the interaction with the host, including organic acids, antimicrobial compounds and enzymes [5]. In addition, cell-envelope related components, such as lipoteichoic acids, exopolysaccharides, or peptidoglycan-derived molecules, have been shown to trigger biological activities attributed to their parent probiotic microorganisms [6,7,8].

By extension, non-viable, generally heat-treated probiotic microorganisms have been shown to exert anti-inflammatory, immunomodulatory, antioxidant, and antimicrobial activities in vitro, ex vivo, and in vivo [9,10]. Jointly known as postbiotics, probiotic subcellular fractions, and non-living preparations are gathering interest both from the scientific and industrial sectors. Due to their intrinsic higher stability in comparison to their living counterparts, postbiotics facilitate technological applications, and supply chain logistics, enabling delivery in challenging food matrices, and extreme climate conditions, where probiotic survival is compromised [11,12,13]. Postbiotics have also shown promise in a variety of clinical settings, including medical foods and infant nutrition [7]. Furthermore, some safety concerns have been raised on the use of live strains in potentially vulnerable groups, such as immune-suppressed patients, again highlighting the interest in exploring the functional effects of postbiotics [14].

*Bifidobacterium longum* CECT-7347 is a probiotic strain isolated from a healthy breast-fed infant, that has been shown in different models to promote anti-inflammatory effects, including the reduction of IFN-γ and TNF-α and increased IL-10 production in human peripheral blood mononuclear cells. Moreover, the reduction of pro-inflammatory markers and increased IL-10 production in monocyte derived dendritic cells, together with an increased expression of zonulin, has also been demonstrated [15,16]. In a clinical trial conducted in children with newly diagnosed celiac disease, typically affected by intestinal inflammation, dysbiosis, and altered intestinal structure, the consumption of the strain resulted in greater height, decreased peripheral lymphocytes, and reduced TNF-α in the CECT-7347 group [17]. In a pilot study on non-celiac gluten sensitivity, the combination of diet and CECT-7347 resulted in the improvement of digestive and extra-intestinal symptoms [18]. Due to its anti-inflammatory/anti-oxidant profile, this strain has also been used in probiotic formulations shown to improve symptomatology derived from atopic dermatitis, psoriasis, and asthenozoospermia, conditions which are related to damaged intestinal integrity [19,20,21,22]. In this context, the purpose of this research is to establish whether the probiotic properties of *B. longum* CECT-7347 are preserved in its heat-treated, non-viable form. Many in vitro and in vivo approaches have been used to characterize the functional effects of probiotics. Among the different animal models available, the small nematode *Caenorhabditis elegans* is particularly interesting because it feeds on bacteria, and is also advantageous given its simplicity, transparency, and ease of cultivation. This nematode has a well-defined anatomy, a rapid reproductive cycle and short lifespan. *C. elegans* is also becoming a genetic model for the molecular identification of many key genes in different cell processes [23]. These advantages have made *C. elegans* an ideal model for characterizing ingredients with antioxidant activity and identifying new antioxidant probiotic strains [24].

Accordingly, we have undertaken a study to evaluate the properties of HT-CECT7347 compared to its parental strain, utilizing *C. elegans* as a model system. Complementary experiments have been performed in HT-29 cells—a human colon cancer-derived cell line extensively used in biological research, for its capacity to form tight monolayers and its similarity to enterocytes, in the small intestine—with the objective of investigating the capacity of *B. longum* HT-CECT7347 to protect against oxidative stress, modulate pro-inflammatory mediators and gut-barrier integrity, together with pathogen colonization. Our study provides further evaluation of postbiotics for digestive and immune health applications.

## 2. Materials and Methods

### 2.1. Bacterial Strains and Culture Conditions

*Bifidobacterium longum* CECT-7347 was isolated from the feces of a healthy infant as described elsewhere [25]. Bacterial cultures were grown in Man–Rogosa–Sharpe (MRS) medium agar and broth supplemented with 0.05% (*w/v*) cysteine and kept at 37 °C in anaerobic conditions for 24 h. For heat inactivation, cells were harvested by centrifugation and washed twice with saline solution and adjusted at stock concentration of 2 × 10^9^ cells/mL. Afterwards, adjusted cultures were treated by autoclaving treatment at 121 °C, 1 bar, during 20 min. *Staphylococcus aureus* (ATCC25923) was cultured on tryptic soy broth (TSB) aerobically. *Salmonella enterica* subsp. *enterica* serovar Typhimurium ATCC 14028 was grown on Luria-Bertani (LB) medium. Both pathogenic strains were grown overnight at 37 °C in aerobic conditions. For *Caenorhabditis elegans* killing assays, overnight cultures were diluted in fresh TSB (*S. aureus*) or fresh LB (*S. typhimurium*) and seeded on NGM agar plates at a final dose of 10^8^ cfu/plate. The plates were incubated aerobically at 37 °C for 18 h.

### 2.2. Cell Cultures

Human colonic epithelial cells (HT-29) were acquired from the American Type Culture Collection (ATCC, Rockville, MD, USA). Cells were cultured as monolayers in a 37 °C humidified atmosphere with 5% CO_2_. The cells were cultured in McCoy’s 5A with 10% heat-inactivated fetal bovine serum (FBS) containing 100 μg/mL streptomycin and 100 IU/mL penicillin.

### 2.3. Cell Treatment and Viability Determinations

HT-29 cells were seeded at 1.5 × 10^6^ cells/well (6-well plates). After complete confluence had been reached, HT-CECT-7347 and TNF-α (4 ng/mL) were added to HT-29 cells in complete medium (McCoy’s 5A, 10% FBS and antibiotics) for 16 h. Cell viability was determined with 3-(4,5-dimethylthiazol-2-yl)-2,5-diphenyltetrazolium bromide (MTT) assay (Abcam).

### 2.4. Expression of Pro-Inflammatory Cytokines

IL-8 concentrations in culture medium were quantified using an enzyme-linked immunosorbent assay (ELISA) kit in HT-29 human colonic epithelial cells, performed according to manufacturer instructions (Abcam).

### 2.5. Western Blotting

For Western Blotting, cells were lysed with lysis buffer (50 mM Hepes, 150 mM NaCl, 1% Triton X-100) including phosphatase and protease inhibitors (1 mM phenylmethylsulfonyl fluoride, 10 μg/mL leupeptin, 10 μg/mL aprotinin, 1 mM sodium ortovanadate, 5 mM NaF, 100 μM TPCK, 1 mM AEBSF, 10 μM E-64, 5 mM phenanthroline, and 20 μg/mL pepstatin) on ice for 20 min. After centrifugation at 12,000× *g* for 20 min, the protein concentration in the supernatant (cytosolic extract) was determined. Pellets were subjected to a second extraction with a nuclear lysis buffer (20 mM Tris-HCl, pH 8, 137 mM NaCl, 1 mM MgCl_2_, 1 mM CaCl_2_, 10% glycerol, 1% NP-40, 0.5% sodium deoxycholate, 0.1% SDS) including phosphatase and protease inhibitors (same as above). Protein samples were subjected to 7.5 or 12% sodium dodecyl sulfate-polyacrylamide gel electrophoresis and transferred to polyvinylidene difluoride membranes (BioRad, Hercules, CA, USA). The membranes were blocked with TBS (10 mM Tris (pH 7.4), 100 mM NaCl, and 0.5% Tween 20) containing 5% bovine serum albumin (BSA) for 4 hours at room temperature and incubated with anti-NFKB/p65 (1:1000, Abcam), zonulin (1:1000, Thermo) or tubulin (1:5000, Abcam) antibodies overnight at 4 °C. Then, the membranes were incubated with horseradish peroxidase (HRP) with conjugated anti-rabbit-immunoglobulin G (IgG) (1:10,000, Abcam). The membranes were visualized by enhanced chemiluminescence (ECL-prime, BioRad) and detected using a Bio imaging system (ImageQuant LAS 500, GE Healthcare). Images were quantified by Image Studio software (LI-COR Biosciences, Lincoln, NE, USA).

### 2.6. Immunofluorescence

Immunofluorescence assays were conducted to analyze the translocation of zonulin, where HT-29 cells were seeded on cover slips at 3 × 10^5^ cells/well (24-well plates). After complete confluence had been reached, HT-CECT-7347, and TNF-α (4 ng/mL) were added to HT-29 cells for 16 h. After treatment, cells were fixed with formaldehyde 4% *v/v* for 10 min at 4 °C and then permeabilized with 0.25% Triton-X100 for 10 min. Next, the cells were blocked with 2% *w/v* bovine serum albumin in PBST + glycine. The samples were incubated with zonulin (5 μg/mL, Thermo) antibodies at 4 °C overnight, followed by incubating with an AlexaFLuor488-conjugated goat anti-rabbit (1:1000, Abcam) for 2 h at room temperature. Fluoroshield medium with DAPI (Abcam) was used for visualization of nucleus. The stained cells were examined by confocal laser scanning microscope (FV1000, Olympus, in the microscopy section of the SCSIE at the University of Valencia).

### 2.7. Caenorhabditis elegans Strains and Maintenance Conditions

*Caenorhabditis elegans* strains N2, Bristol (wild-type) and the mutant strain GR1307, *daf-16* (*mgDf50*) were obtained from the *Caenorhabditis* Genetics Center at the University of Minnesota and maintained at 20 °C on Nematode Growth Medium (NGM) plates with *Escherichia coli* strain OP50 as normal diet for nematodes.

### 2.8. Lifespan Assays in C. elegans

To measure the lifespan of *C. elegans*, synchronized worms of the wild-type strain (N2) and the mutant strain GR1307 (daf-16) were grown at 20 °C until they reached the young adult stage. Worms were then transferred to NGM agar plates covered with lawns of *E. coli* OP50 or *B. longum* CECT-7347. For heat-treated CECT-7347, culture was adjusted and added to the agar surface at three final doses (10^8^, 10^9^, 10^10^ cells/mL). The plates were incubated at 20 °C and the numbers of live and dead worms were scored until 100% population was dead. Two independent assays were carried out with each cell concentration and strain.

### 2.9. Oxidative Stress Resistance in Caenorhabditis elegans

*Caenorhabditis elegans* wild-type strain N2 and the mutant strain GR1307, *daf-16* (mgDf50) were egg-synchronized in NGM plates (control medium) and NGM plates containing CECT-7347 or HT-CECT-7347 at final dose of 10^8^ cells/plate. Nematode viability was assessed after oxidative stress (2 mM H_2_O_2_) as described elsewhere [26]. Vitamin C (10 µg/mL) was used as positive control. Experiments were carried out in duplicate.

### 2.10. Infection Assays in C. elegans

Infection assays were performed as described elsewhere, with some modifications [27,28,29]. Nematodes of the wild-type strain (N2) were age-synchronized by recovering the eggs from adults in agar plates, already seeded with *E. coli* OP50 (NGM), and NGM plates containing either CECT-7347 or HT-CECT-7347, at a dose of 10^8^ cells/plate. Once the worms reached the young adult stage, they were transferred to the infection plates containing a lawn of the pathogen (*S. aureus* ATCC 25923 or *S. enterica* subsp. *enterica* serovar Typhimurium ATCC 14028). A condition without infection (NGM medium with the strain *E. coli* OP50) and a condition of infection only with the corresponding pathogen were included. Afterwards, nematodes were incubated at 25 °C in the different conditions and scored for survival during 5–10 days. Worms were counted as alive or dead individuals by gentle touching with a platinum wire.

### 2.11. Evaluation of Gut Barrier Integrity in C. elegans

Age-synchronized nematodes of the wild-type strain N2 were obtained and maintained in NGM plates or NGM plates supplemented with the strain CECT-7347 or HT-CECT-7347 strain (10^7^ and 10^8^ cells/plate) at 20 °C. To induce intestinal permeability L4-larvaes were exposed to methotrexate (MTX 0.1 µg/mL) for 24 h (acute exposure). A condition without damage was also included. To evaluate the intestinal permeability in MTX exposed-nematodes, Nile Red staining (0.05 µg/mL) was used [30]. A total of 30 worms were randomly selected from each condition and observed in a fluorescence stereomicroscope Nikon SMZ18, equipped with NIS-ELEMENT image software. Results are shown as the percentage of fluorescence in each treatment with respect the MTX-treated nematode population.

### 2.12. Statistical Analysis

All the statistical analyses were performed with GraphPad Prism 4 software. Data were expressed as the mean ± standard deviation and analyzed by one-way ANOVA, using a Tukey’s multiple comparison post-hoc test. A probability value of 0.05 was considered statistically significant. Survival curves were analyzed using the log Rank T-test significance test, provided by GraphPad Prism 4.

## 3. Results

### 3.1. Antioxidant Effect of Bifidobacterium Longum CECT-7347 and Its Heat-Treated Version, in Caenorhabditis elegans

**Figure 1:**
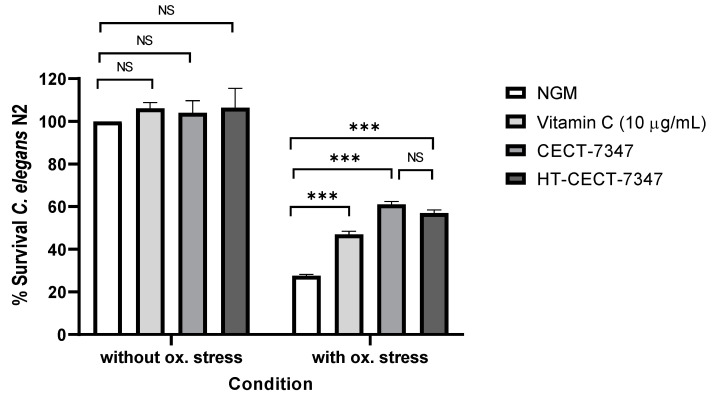
Survival of *C. elegans* (N2) fed with *B. longum* CECT-7347 or HT-CECT-7347 without oxidative stress and after acute oxidative stress. NGM: Nematode growth medium used as control feeding condition. *** Significant *p* < 0.001; NS: No significant differences. One-way ANOVA was applied. Data are the average of two independent experiments (*n* = 200/condition).

Probiotic strains (live and heat treated cells) exert antioxidant activity, reducing damage caused by oxidative stress [31,32,33,34]. Moreover, previous studies have demonstrated that probiotics strains are able to exert antioxidant effect on the nematode *C. elegans* [35,36,37]. Therefore, to confirm the antioxidant properties of the heat-treated *B. longum* HT-CECT-7347 strain, while comparing them to those exerted by the viable CECT-7347 strain, we used the in vivo model *C. elegans*. Nematodes of the wild-type N2 strain were fed with the heat-treated CECT-7347 or the live cells, and their survival rate after acute oxidative stress with hydrogen peroxide was compared to control-fed nematodes (NGM with *E. coli* OP50). As reference, survival of nematodes without oxidative stress was included. Results indicate a similar survival under the different feeding conditions in non-stressed nematodes. However, nematodes treated with strain HT-CECT-7347 were more resistant to oxidative stress with H2O2 than control-fed nematodes (57% vs. 27.5% of survival value) (*p* ≤ 0.001) (Figure 1). Moreover, this activity was similar to that observed in CECT-7347 live cells, thus demonstrating that the heat-treated cells still preserve the ability to protect in vivo against acute oxidative stress.

**Figure 2:**
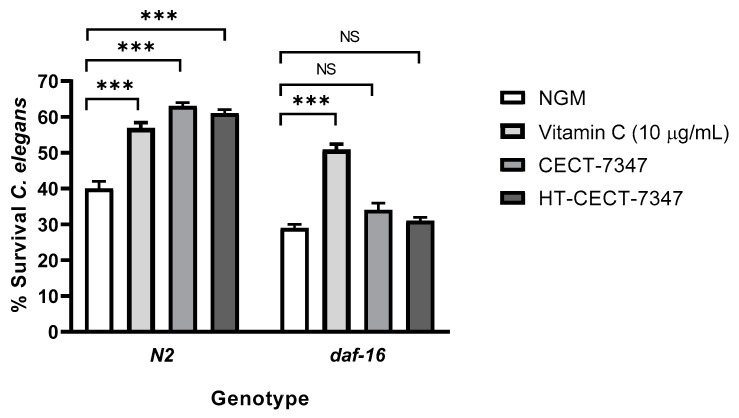
Survival of *C. elegans* mutant strain in DAF-16 fed with *B. longum* CECT-7347 or HT-CECT-7347 after acute oxidative stress. NGM: Nematode growth medium used as control feeding condition. *** Significant *p* < 0.001; NS: No significant differences. One-way ANOVA was applied. Data are the average of two independent experiments (*n* = 200/condition).

Furthermore, to examine the mechanisms underlying this functional effect, we repeated the experiments in a *C. elegans* mutant strain, *daf-16*. This strain was fed with CECT-7347 and HT-CECT-7347 and subjected to acute oxidative stress. DAF-16 codes for the Forkhead family of transcription factors (FOXO) and plays a central role in mediating the molecular mechanisms triggered by the insulin-like signaling pathway (IIS). This pathway regulates ageing, immunity, and lipid metabolism in *C. elegans* [38,39]. Results indicated that feeding DAF-16 mutant worms with HT-CECT-7347 or CECT-7347 did not increase worm survival rates, suggesting that the mechanism of the strain (both live and heat-treated cells) is dependent on DAF-16 (Figure 2). By contrast, vitamin C activity was independent of DAF-16, as significant survival was observed in the mutant strain.

Previous studies have correlated a strengthened resistance to oxidative stress in *C. elegans* with lifespan extension in response to the consumption of probiotic strains [35,40]. *Caenorhabditis elegans* represents a suitable model organism for ageing studies as it can be easily grown on agar plates, fed with bacteria or yeast, and its lifespan is modulated by genetic, and environmental factors [41]. Moreover, genetic pathways such as IIS [42] and p38 mitogen-activated protein kinase (p38 MAPK) [43] pathways involved in life extension are evolutionarily conserved. Indeed, different probiotic strains have been shown to have pro-longevity effects in the nematode [44].

**Figure 3:**
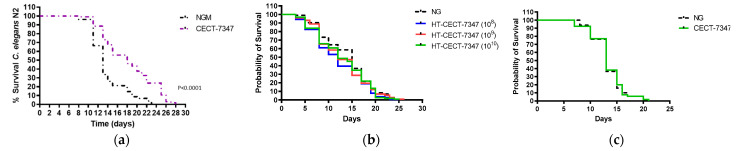
Effect of the strain CECT-7347 and HT-CECT-73 47 on *C. elegans* lifespan. (**a**) *B. longum* CECT-7347 significantly increases lifespan of *C. elegans* wild-type strain (N2) (**b**) Cells of HT-CECT-7347 do not increase *C. elegans* (N2) lifespan (dose 10^8^, 10^9^, and 10^10^ cells/plate) (**c**) Lifespan increase by *B. longum* CECT-7347 is lost in the *C. elegans* in DAF-16 (GR1307) mutant strain. Curve comparisons vs. *E. coli* OP50 are indicated (*p*-values). Log Rank T-test was applied. Data correspond to the average of two independent assays (*n* = 200/condition).

To gain a better understanding of the properties of the strain and its heat-treated counterpart, we analyzed the effect of both CECT-7347 and HT-CECT-7347 on *C. elegans* lifespan. As shown in Figure 3a, the living strain significantly improved lifespan (*p* < 0.0001), and an increase of 5–6 days in mean lifespan was observed compared with control NGM. Conversely, the heat-treated form HT-CECT-7347, preserved, but did not extend, worm’s viability. This result indicates that this form of the strain is not harmful for *C. elegans*. Additionally, the results would suggest a mechanism for lifespan extension dependent on cell viability, is probably related to the production of a specific metabolite.

Furthermore, we analyzed whether the CECT-7347-related increase in lifespan was dependent on DAF-16. It is well known that *daf-16* is a regulator of the inflammatory response, via a pathway that is well conserved in humans [35]. Therefore, we evaluated the effect of CECT-7347 on *C. elegans daf-16* mutant strain. A loss of the protective effect in the *daf-16* mutant was determined (Figure 3b), suggesting that the pro-longevity effects of CECT-7347 are dependent on the *daf-16* pathway.

### 3.2. Anti-Inflammatory Properties of B. Longum HT-CECT-7347 in HT-29 Cells: Suppressive Effect on Pro-Inflammatory Cytokines and NF-κB Activation

Probiotics capable of alleviating oxidative stress in *C. elegans*, via the DAF16 transcriptional factor, have been shown to present an anti-inflammatory profile when co-cultured with HT-29 cells, stimulated by pro-inflammatory cytokines [35]. Therefore we hypothesized that, owing to its anti-oxidant characteristics, heat-treated CECT-7347 could down-play the production of pro-inflammatory cytokines upon TNF-α stimulation. TNF-α triggers inflammatory responses and develops inflammation by inducing the expression of pro-inflammatory cytokines, including IL-1β, IL-6, and IL-8.

**Figure 4:**
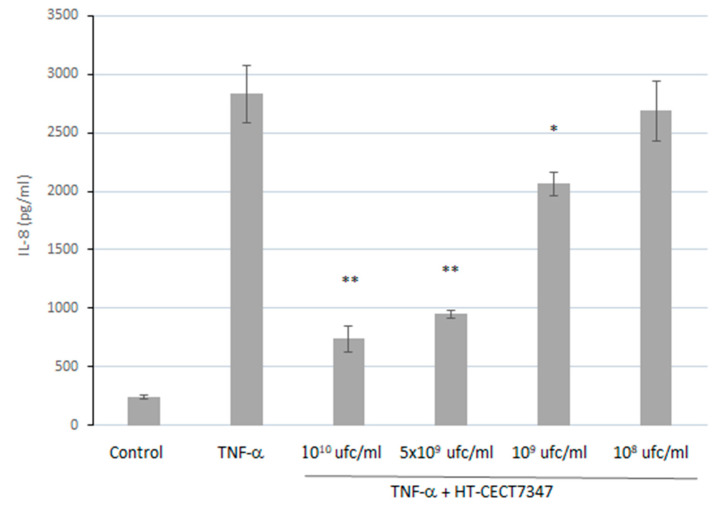
Quantification of IL-8 secretion by HT-29 cells incubated with HT-CECT-7347 and stimulated with TNF-α. Data are given as means and standard deviation (*n* = 2). *, ** indicates significantly lower than TNF-α control (*p* < 0.05 and *p* < 0.01, respectively).

**Figure A1:**
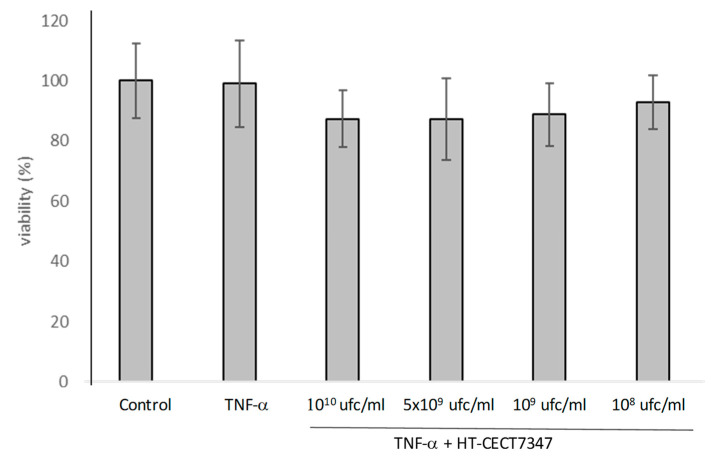
Maintenance of cell viability upon TNF-α stimulation in the different samples in which IL-8 production has been quantified, corresponding to Figure 4 in the main text.

The human epithelial cell line HT29 has previously been described as having the ability to produce the chemokines IL-8, Groα, macrophage inflammatory protein 1, and RANTES upon stimulation with IL-1, TNF-α, and interferon γ [45,46]. In order to assess the anti-inflammatory properties of HT-CECT-7347, IL-8 production was evaluated by incubating HT29 cell monolayers with TNF-α in the presence of HT-CECT-7347. As shown in Figure 4, the stimulation of HT29 cells with TNF-α followed by co-incubation with HT-CECT-7347 resulted in a significant dose-dependent decrease in IL-8 production, without significantly affecting its viability (Figure A1). Concentrations equivalent to 10^10^ and 10^9^ cells/mL of HT-CECT-7347 reduced the secretion of IL-8 by 73.8% (743 ± 111 pg/mL) and 27.0% (2066 ± 101 pg/mL), respectively. 

Moreover, NF-κB plays a key role in regulating cytokine expression levels following a proinflammatory stimulus [45]. In the absence of stimulus, NF-kB is maintained in its inactive form in the cytosol associated with the inhibitory protein IkB. Upon receipt of a proinflammatory stimulus, such as TNF-α, translocation of several adapters to the cytoplasmic membrane occurs, such as TNF receptor-associated death domain protein (TRADD), receptor-interacting protein (RIP) and TNF receptor-associated factor 2 (TRAF2), which activates the IkB-kinase (IKK) complex [46]. NF-kB p50-p65 heterodimer proteins are released after phosphorylation of inhibitory kB (IkB) molecules by IKK, migrate to the cell nucleus and bind to specific kB sites, leading to transcriptional activation of genes coding for cytokines and chemokines, cell adhesion molecules, and immunoreceptors, all of which are important mediators of the inflammatory response [47,48,49,50].

**Figure 5:**
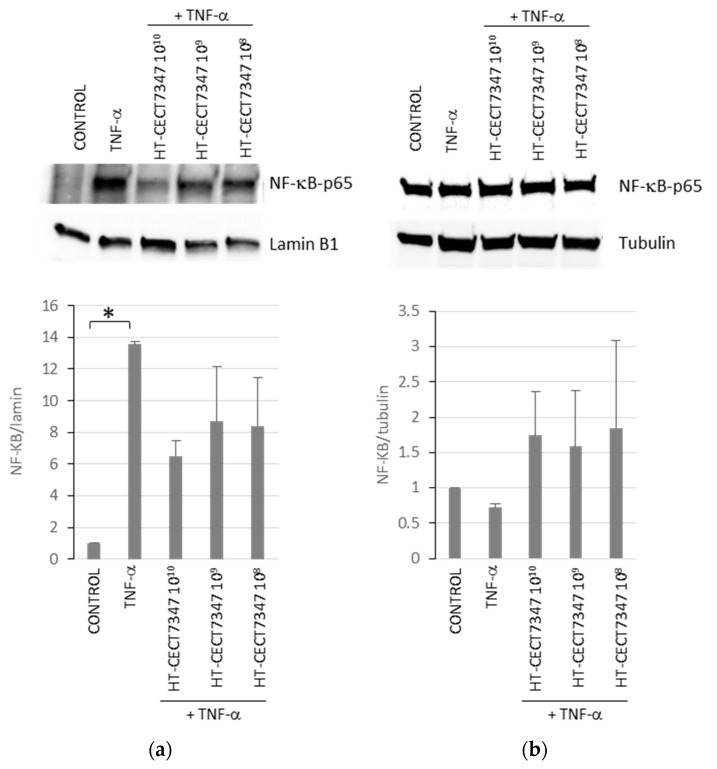
Analysis of the effect of HT-CECT-7347 on TNF-α-induced nuclear translocation of NF-kB p65 subunit in HT-29 cells. Western blot analysis for NF-kB-p65 subunit levels in nuclear (**a**) or cytosolic (**b**) fractions of HT-29 cells treated with TNF-α with or without HT-CECT-7347. Expression results were quantified by Image Studio software (LI-COR Biosciences) and represented as a graph. Data represents the means (+/− SD) of two independent experiments. * indicates significantly higher than control (*p* < 0.05).

Therefore, we evaluated whether the inhibitory effects of HT-CECT-7347 on the pro-inflammatory cytokines were due to its ability to suppress TNF-α-induced NF-κB activation. With this purpose, we examined NF-kB-p65 subunit localization after HT-CECT-7347 treatment in TNF-α -induced HT-29 cells by Western blot, results shown in Figure 5. Compared to the TNF-α individual treatment, HT-CECT-7347 and TNF-α co-treatment decreased NF-kB-p65 nuclear localization and increased NF-kB-p65 expression in the cytosol.

### 3.3. Gut Barrier Protection and Inhibition of Bacterial Colonization Capacity

Epithelial barrier integrity and exchange between bloodstream and intestinal lumen is regulated by tight junctions (TJ), protein structures that constitute the major barriers sealing paracellular spaces between epithelial cells. Zonulin, a protein released by intestinal epithelial cells, acts as a TJ modulator. Probiotic strains have been shown to exert beneficial effects on intestinal barrier function, modulating tight junction proteins and zonulin expression [51,52]. Previous studies have reported the beneficial effect of CECT-7347 on intestinal permeability [15]. Since gut-barrier disruption can lead to endotoxemia, triggering an inflammatory response and resulting in increased susceptibility to infection, we investigated whether CECT-7347 and HT-CECT-7347 could provide a protective effect on TJ, while inhibiting bacterial colonization.

**Figure 6:**
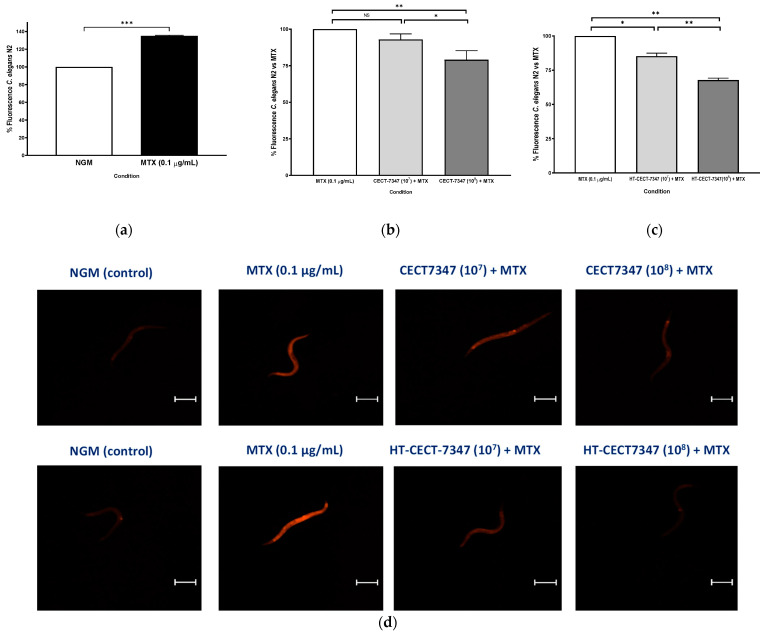
(**a**) Percentage of fluorescence intensity of Nile Red in worms without gut damage (NGM condition) or in worms with MTX-induced intestinal damage at dose of 0.1 µg/mL. Data are the average of two independent experiments (*n* = 60/condition); (**b**) Percentage of fluorescence of worms treated with MTX at 0.1 µg/mL (gut damage condition) with CECT-7347 or (**c**) with HT-CECT-7347 at two different doses (10^7^ and 10^8^ cells) + MTX (0.1 µg/mL). Values are the average of two independent assays. (*n* = 60/condition). *** Significant at *p*-value ≤ 0.001. ** Significant at *p*-value ≤ 0.01; * Significant at *p*-value ≤ 0.05. NS: No significant differences. One-way ANOVA was applied. (**d**) Representative images of Nile Red staining in live young adult *C. elegans* in a wild-type N2 animal under fluorescence microscopy. Worms treated with MTX to induce intestinal damage were fed with CECT-7347 or HT- CECT-7347 strain at two doses (10^7^ and 10^8^ cells). Scale bar 250 µm. Original image taken by the authors for this paper with a Nikon-SMZ18 fluorescence stereomicroscope.

To investigate their efficacy in preventing leaky gut, worms were fed with CECT-7347 and HT-CECT-7347, and treated with MTX to alter nematode intestinal permeability. Drugs with MTX have been used to treat autoimmune diseases and cancer, and their intestinal toxicity has been described in humans and mice (Huang et al., 2020). Firstly, we determined that MTX-exposed nematodes significantly enhanced fluorescence intensity of Nile Red compared to control nematodes (NGM plates without MTX) (Figure 6a) (increase of 35.1%) showing an alteration in intestinal permeability in *C. elegans*, as previously reported for other toxins [53] and also shown for MTX in mice and rats [54,55]. [54,55]. By contrast, pre-treatment with the heat-treated probiotic HT-CECT-7347 or CECT-7347 blocked the increase in fluorescence intensity by Nile Red staining (Figure 6b). Representative images of nematodes in each condition, obtained by fluorescence microscopy, are shown in Figure 6d. A significant reduction in the fluorescence intensity at both doses analyzed (10^7^ and 10^8^ cells) fed with HT-CECT-7347 was observed in MTX-treated nematodes, being 10^8^ CFU dose the most effective (32.1% reduction, *p*-value ≤ 0.01). In the case of the live cells, only the dose of 10^8^ cells was significant in reducing fluorescence. These results would suggest that HT-CECT-7347, and to a lesser extent CECT-7347, help in maintaining intestinal permeability in MTX-exposed nematodes.

**Figure 7:**
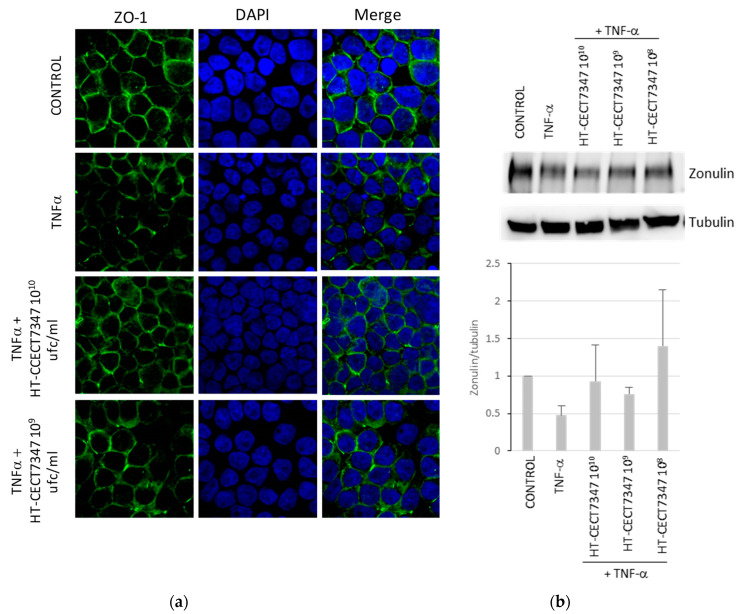
Analysis of the effect of HT-CECT-7347 on zonulin localization on TNF-α-stimulated in HT-29 cells. (**a**) Representative images from immunofluorescence analysis of zonulin (green fluorescence AlexaFluor 488) in TNF-α stimulated cells show a significant decrease in staining of ZO-1. Localization of ZO-1 is not affected when cells are stimulated by TNF-α in the presence of HT-CECT-7347. Micrographs were captured at 60× magnification. (**b**) Western blot analysis for zonulin levels in cell extracts of HT-29 cells treated with TNF-α with or without HT-CECT-7347. Expression results were quantified by Image Studio software (LI-COR Biosciences) and represented as a graph. Data represents the means (+/− SD) of two independent experiments.

In order to confirm a TJ-mediated effect, zonulin (ZO-1) in HT-29 monolayers was analyzed with immunofluorescence to determine whether *B. longum* HT-CECT-7347 could affect the localization and level of zonulin on the apical surface of adjacent HT-29 cell membranes during inflammation. As shown in Figure 7a, immunofluorescence analysis of control cells showed the presence of a continuous line at the contact points of the cells, which was fragmented after treatment with TNF-α. Conversely, when cell cultures were co-treated with HT-CECT-7347 and TNF-α, immunofluorescence showed a pattern similar to that of the control. Moreover, Western blot analysis revealed that HT- CECT-7347 counteracted the TNF-α-induced hypoexpression of ZO-1 (Figure 7b).

To further explore the potential of the strain in the context of digestive malfunction, we investigated whether CECT-7347 and/or HT-CECT-7347 were protective against bacterial infection. *Caenorhabditis elegans* has been reported as a host for several human pathogens such as *S. enterica* subsp. *enterica* serovar Typhimurium and *S. aureus* [56,57,58] and it is being used as an alternative to traditional mammalian pathogenesis models [59]. *Salmonella* causes persistent infection in the intestine of *C. elegans*, resulting in early death of infected animals, while *S. aureus* does not persistently colonize the digestive tract of nematodes but ultimately leads to worm death [58]. We explored whether CECT-7347 and HT-CECT-7347 increased *C. elegans* defenses against pathogens by exposing them to the probiotic cells in agar-based killing assays with *Salmonella or S. aureus*.

**Figure 8:**
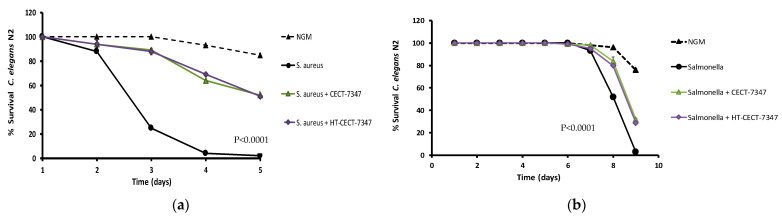
Effect of CECT 7347 and HT-CECT 7347 on resistance of *C. elegans* to *S. aureus* and *S. enterica* subsp. *enterica* serovar Typhimurium. (**a**) Worms fed with *E. coli* OP50 (control), *S. aureus*, and CECT 7347 or HT-CECT 7347 in the presence of the pathogen. Worms fed with *B. longum* cells (both live and heat-treated cells) were more resistant to infection (*p* < 0.0001) (**b**) Worms fed with *E. coli* OP50 (control), *S. enterica* subsp. *enterica* serovar Typhimurium, and CECT-7347 or HT-CECT 7347 in the presence of the pathogen. Worms fed with *B. longum* cells (both live and heat-treated cells) were more resistant to infection (*p* < 0.0001). Log Rank T-test was applied. Data are the average of two independent experiments (*n* = 100/condition).

As shown in Figure 8a, *S. aureus*-infected nematodes under control-feeding condition reduced viability in a few days, reaching 80% mortality at day 3, and 100% at day 5. Conversely, nematodes fed with the probiotic CECT-7347 or its heat-treated form HT-CECT-7347 significantly increased in survival (*p* < 0.0001), maintaining around 70% of survival at day 3 and 50% of survival at day 5. In the case of *S. enterica* subsp. *enterica* serovar Typhimurium CECT 4594, results indicated the efficacy of the pathogen to infect and provoke mortality in *C. elegans* during days 6 and 9 of infection (Figure 8b). The exposure of nematodes to CECT-7347 or HT-CECT-7347 resulted in a significant increase in viability (*p* < 0.0001), with 80% of survival at day 8 (vs. 50% in control-fed nematodes), and 70% of survival (vs. 100% in control conditions).

## 4. Discussion

Disrupted intestinal barrier function triggers an inflammatory response, as increased permeability leads pathogens and toxins into tissues and blood stream, causing endotoxemia. Conversely, systemic inflammation impairs the epithelial barrier, cytokines and interleukins activating NF-κB signaling pathway that in turn induces TJ disassembly. These processes are typically characterized by oxidative stress, with excessive ROS production as host defense is weakened, and by markers of intestinal barrier damage, redox imbalance, and inflammation [60,61].

Probiotic strains have been reported to help maintain the homeostasis of intestinal function, by regulating the expression of TJ proteins at cell boundaries, reducing adverse effects of pathogens, and protecting them from oxidative stress-induced damage, by scavenging ROS and/or preventing their formation while modulating inflammatory cascades [9,31,34]. Similarly, beneficial properties have been documented in non-viable probiotics, including anti-inflammatory, anti-pathogen adhesion, and gut barrier function attributes [6,10,14,62].

However, few studies report on protection against oxidative stress in postbiotics [31,33]. Indeed, few subcellular postbiotic fractions or heat-treated probiotic strains have been shown to exert antioxidant effects [14,37,63]. References for antioxidant postbiotics derived from *Bifidobacteria*, a genus of substantial ecological significance for digestive well-being, are even more limited [33,64,65].

In this study, we have demonstrated how both living and heat-treated *B. longum* CECT-7347 cells resulted in higher survival rates of *C. elegans* following acute oxidative stress, while the live form increased lifespan. Interestingly, both effects seem mediated by DAF-16, the Forkhead family of transcription factors (FOXO) downstream insulin signaling pathway (IGF-1). In *C. elegans*, this is one of the main pathways involved in lifespan control, oxidative stress, regulation of immune response and defense against pathogen infection [66]. These results further validate the previous report by Sugawara and coworkers, on a heat-killed *B. longum* strain, also suggesting a DAF-16 mechanism, independent of SKN-1 [37]. This observation contradicts previous findings for other probiotic species, describing a SKN-1-mediated, DAF-16-independent, mechanism [40,67]. Further analysis should be performed in order to validate alternative molecular modulators of DAF-16, such as the JNK pathway or parallel pathways like SKN1 via p38 MAPK pathway.

**Figure A2:**
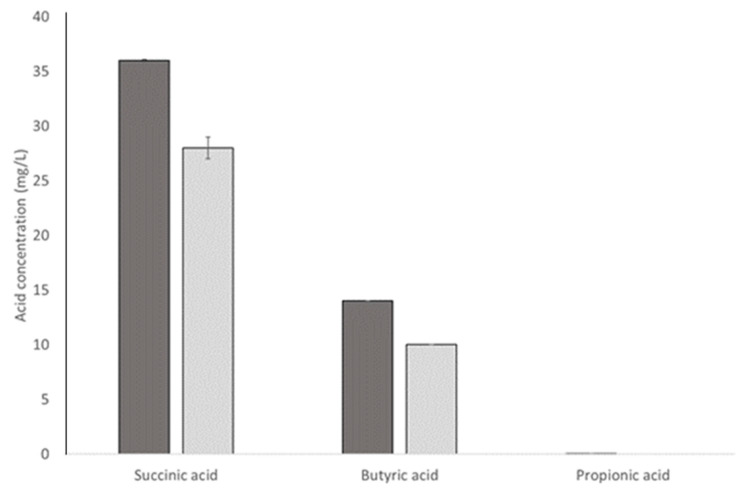
Succinic, butyric and propionic acid production by *B. longum* CECT7347, determined by HPLC in culture supernatant in MRS medium, upon 24 and 48 h of growth.

Furthermore, heat-treated CECT-7347 did not result in improved lifespan, unlike living CECT-7347. Further research should investigate the precise mechanism by which the living form, but not HT-CECT-7347, contributes to longevity and anti-aging properties in the worm model. However, it is worth noting that CECT-7347 is a butyric acid producer (Figure A2). Short-chain fatty acid (SCFA) butyric acid has been shown to extend *C. elegans* lifespan; therefore, we can hypothesize that this particular trait, which is unlikely to be preserved in the heat-treated form, contributes to the differential lifespan of CECT-7347-fed *C. elegans*.

Following previous observations in probiotic strains correlating anti-inflammatory phenotype with the capacity to alleviate oxidative stress in *C. elegans* through the DAF16 transcriptional factor, we investigated whether co-incubation with HT-CECT-7347 could attenuate the release of inflammatory IL-8 upon TNF-α stimulation. Multiple studies have shown the impact of probiotic bacteria by attenuating IL-8 secretion in cytokine-stimulated cells, thus potentially contributing to the maintenance of intestinal homeostasis [68,69,70,71,72]. NF-κB is a key regulator of the inducible expression of many genes involved in immune and inflammatory responses in the gut [73]. Its activation is strongly induced in the inflamed gut, with levels correlating significantly with the severity of intestinal inflammation [74]. Furthermore, in mammals, there is a direct link between signaling via longevity factors, such as FoxOs, and inhibition of NF-kB signaling [75]. Accordingly, our findings show a dose-dependent decrease in IL-8 production, mediated by the impaired NF-kB nuclear localization as a result of HT-CECT-7347 feeding. Therefore, according to our data, HT-CECT-7347 has the potential to protect intestinal cells from an acute inflammatory response.

Protection against pathogens has also been described in heat-killed bacteria, mostly in *Lactobacilli*, capable of reducing the effects of the infection of pathogens such as enterotoxigenic *E. coli*, *Salmonella*, *Campylobacter*, and *H. pylori*, by enhancing the immune response and competing for gastrointestinal adhesion sites [14]. However, reports of inactivated bifidobacteria leading to enhanced resistance to pathogens are scarce [76,77]. In this study, we have shown how both living and heat-treated CECT-7347 reduce the lethality of both Gram positive (*Salmonella*) and Gram negative (*S. aureus*) pathogens in *C. elegans.* These results, together with its observed ability to increase longevity in a DAF-16 dependent-manner, would suggest an immune modulatory activity of the strain [78]. Whether this occurs by competitive exclusion of pathogen adhesion, and/or enhancement of innate defense mechanisms, has yet to be investigated.

As previously mentioned, in the context of inflammation and oxidative stress, support of TJ integrity is relevant to prevent or restore impaired gut barrier function. Interestingly, in a dose-dependent manner, HT-CECT-7347 was able to maintain the intestinal permeability upon MTX disruption at higher levels than its living counterpart CECT-7347. The toxic effects of MTX, a folate antagonist widely used in the treatment of cancer and autoimmune diseases, are well described, and include intestinal toxicity [79]. To ameliorate the secondary effects, different studies suggest the use of a reduced form of folate derivate, Leucovorin (LV), as a treatment to counteract the intestinal toxicity provoked by MTX (Huang et al. 2020). Bearing in mind the important role played by the microbiome on intestinal integrity, other approaches are based on the use of probiotics of *Bifidobacterium* genera, which can produce large amounts of active metabolites, including folate. We hypothesized that the production of a metabolite, like folate, could be responsible of the protective effect of strain *B. longum* CECT-7347 in *C. elegans* MTX-induced leaky gut. However, the fact that a greater effect was observed with the heat-treated strain would suggest that the underlying mechanism of action is a biomass component of the probiotic strain, rather than its metabolic activity. The effects have been correlated with increased zonulin expression, in agreement with previous findings for the live CECT-7347 strain [15]. Furthermore, our results also agree with observations of other *B. longum* strains, which report reduced MTX-induced intestinal damage in mice through decreased inflammation, as well as a reduction in Zoo-1 expression in intestinal tissue (Huang et al. 2020). Gut barrier integrity has been shown as a key factor, cause or consequence, in multiple conditions involving gut inflammation in their clinical presentations. Altered gut permeability has been linked with fibromyalgia, chronic fatigue, neurodegeneration processes [80,81,82], arthritis [83], and metabolic disorders [84], among others. Furthermore, skin alterations have also been linked with gut permeability, correlating with disease severity in the case of psoriasis [85]. Furthermore, in some of the studies, restoration of the gut barrier was also linked to clinical improvement [80,83,84]. In this context, ingredients preserving intestinal permeability have the potential to positively contribute to well-being in conditions with underlying inflammatory digestive origin. In this context, CECT 7347 and HT-CECT 7347 may have a significant role to be further investigated in future studies.

## 5. Conclusions

In summary, this study investigated the properties of HT-CECT-7347, the heat-treated form of probiotic strain CECT-7347, and compared it with its live form. Results highlight its capacity to counter-balance oxidative stress damage, together with other stressors related to intestinal inflammation, including pro-inflammatory mediators and gut-barrier disruptors. The results obtained are consistent with the anti-inflammatory, gut-barrier protective effects previously described for the live strain [15,25]. The fact that most of the functional properties of this strain are preserved in the heat-treated form provides further information on postbiotics, supporting the notion that non-*Lactobacilli* probiotic strains can remain biologically active despite being non-viable. Future research will explore the specific mechanisms through which HT-CECT-7347 exerts its functionality, and also investigate its effects in higher animal models, targeting immune function support and gut health. The availability of heat-stable postbiotics, such as HT-CECT-7347, is essential to broaden the range of applications of functional microbial solutions, enabling delivery in challenging matrixes and facilitating logistics in extreme environmental conditions.

## Data Availability

The data presented in this study is contained within the article.
